# Peer Adoption and Development of Health Innovations by Patients: National Representative Study of 6204 Citizens

**DOI:** 10.2196/11726

**Published:** 2019-03-26

**Authors:** Pedro Oliveira, Leid Zejnilovic, Salomé Azevedo, Ana Maria Rodrigues, Helena Canhão

**Affiliations:** 1 Copenhagen Business School Copenhagen Denmark; 2 Católica-Lisbon School of Business and Economics Lisbon Portugal; 3 Nova School of Business and Economics Nova University Lisbon Portugal; 4 CEDOC, EpiDoC Unit, Nova Medical School, Nova University Lisbon Portugal; 5 Associação EpiSaúde Évora Portugal; 6 Faculdade de Medicina da Universidade de Lisboa Lisbon Portugal; 7 National School of Public Health, Nova University, Lisbon, Portugal Lisbon Portugal

**Keywords:** citizen, patient, innovation, therapeutics, health, development, adoption, physician-patient relations, social interactions, search engine

## Abstract

**Background:**

There is growing evidence that many patients and caregivers innovate by developing new solutions to cope with their health disorders. Given the easy access to vast internet resources and peers globally, it is increasingly important to understand what may influence user innovation and its adoption in health for improving individual well-being and ensuring their safety, in particular, how interactions with peers and physicians or search behavior, along with sociodemographics, may influence the decision to develop a solution or adopt one developed by a peer.

**Objective:**

The aim of this paper was to study the development and peer-to-peer adoption of user innovations in health care and identify individual-level factors associated with these processes.

**Methods:**

Data were collected via computer-assisted phone survey from a large, random, and representative sample of adult residents in Portugal (N=6204). User innovation questions were added to 1 wave of an ongoing observational, longitudinal, population-based epidemiological study. By asking about individual innovation activity, the sample was split into 3 groups: (1) the developers of health-related solutions for own use (developers), (2) the adopters of solutions developed by other patients or caregivers (peer-to-peer adopters), and (3) the rest of the population. Within the last group, *intention to adopt* was measured and used as a proxy of future behavior. Regression analysis is used to test the associations.

**Results:**

In the population considered in this paper, an estimated 1.3% (75/6008) reported having developed a solution for own use and 3.3% reported to have adopted a solution developed by peers. The 3 groups (developers, adopters, and remaining population) have distinctive characteristics. Gender plays an important role in the solution development, as women are less likely to develop one (odds ratio [OR] 0.4, 95% CI 0.20-0.81; *P*<.05). Education is positively associated with the development activity (OR 1.13, 95% CI 1.03-1.24; *P*<.05) but also with the intentions to adopt a peer-developed solution. Search for health-related information is positively associated with the development, adoption, and the intentions to adopt a solution. Interactions with peers over the internet are rare, but in-person interactions are frequent and have a positive association with the dependent variables in all 3 groups. The results also suggest that trust in doctors represents an important dimension that shapes the attitudes of the population toward peer-developed solutions.

**Conclusions:**

This paper demonstrates the importance of the peer community, doctor-patient relationship, citizen’s search for information on innovation, and individual attitudes toward peer-to-peer adoption in health care. It stresses the need for a reliable Web-based health-related information and the necessity to deeper understand complex relationships between the need to improve health and fulfill the need and the perception of the health care system.

## Introduction

### Background

User innovators are the ones who have developed a new good or service or modified an existing good or service for own use; they differ from producer innovators for whom profit is the dominant motivation to innovate [1]. Innovation scholars have demonstrated the existence of this empirical phenomenon in numerous industries, including health care [2]. Survey evidence from measurement studies of user innovation at a national level estimated that up to 0.5% of citizens in the United States, Japan, Finland, and the United Kingdom modify or create new products and services for personal health care–related use [3,4].

The largest group of user innovators in health care are *patient innovators*, patients or their nonprofessional caregivers (eg, parents and family members), who modify or develop a treatment, a technical aid product, or a medical device to cope with a health condition [5]. Besides developing, they may also share or adopt solutions developed by other peers, they organize themselves in communities, and either individually and or jointly solve problems, and even do limited trials with solutions they develop [6-9]. A study conducted in a population of rare disease patients and their nonprofessional caregivers showed that the frequency of user innovation might be higher among those afflicted with rare diseases than in the general population. The authors reported that 36% of interviewed survey respondents had developed a solution that was new to them, and 8% of the interviewed survey respondents had developed solutions that may be novel to the medical practice [6].

When the value of patient-developed solutions is considered, there is evidence of patients reporting significant improvements in the quality of life after using their self-developed solutions [6]. Furthermore, the study by Oliveira and Canhão [10] identified successful cases of novel patient-developed solutions that made a significant impact on medical practice. Health benefits and social value of innovations can only be achieved when innovations diffuse—when they are adopted and used by other people. In national-level surveys, the fraction of diffused user innovations observed varied from 5% to 17%, with the most common diffusion pathway being peer-to-peer exchange [3,4,11]. In the rare disease study, 32% of solutions reported by the patients and caregivers were shared with others—almost double the highest diffusion incidence observed in the general population—but only 5% shared the information with their doctor [6]. The existing evidence strongly suggests that the innovation and diffusion activity by patient innovators is significant but mostly hidden from the traditional health care system. The adoption side of the peer-to-peer innovations in health has received little or no attention from academia.

### Rational and Aim

Innovation and adoption activity by patients and caregivers in health care may be strongly influenced by health care–related and sociotechnological contextual factors. However, no prior work systematically explored such relationships. For example, we know that people invest significant efforts to search for health-related information (online and offline) [12] and may have well-developed strategies for evaluating the credibility of the information [13]. However, we do not know the relationship of such a search with the innovation or peer-to-peer adoption activity. Other sociotechnical and health care contextual factors of interest may also include characteristics of the peer networks among patients [14,15], doctor-patient relationships [15-17], personal responsibility for health, or the trust in the availability of scientific breakthroughs for their health disorder. This work is, to the best of our knowledge, the first to systematically explore the relationships between these contextual factors and health-related peer-to-peer innovation and adoption activity among citizens.

## Methods

### Research Design

The data used in this paper are survey responses from a random and representative sample of adult residents in Portugal (N=6204), collected via computer-assisted phone survey conducted by NOVA Medical School. Professional interviewers were additionally trained by a psychologist to communicate the innovation questions, and 2 of the authors trained them to fill in the survey responses in a computer program during the conversation.

**Figure 1:**
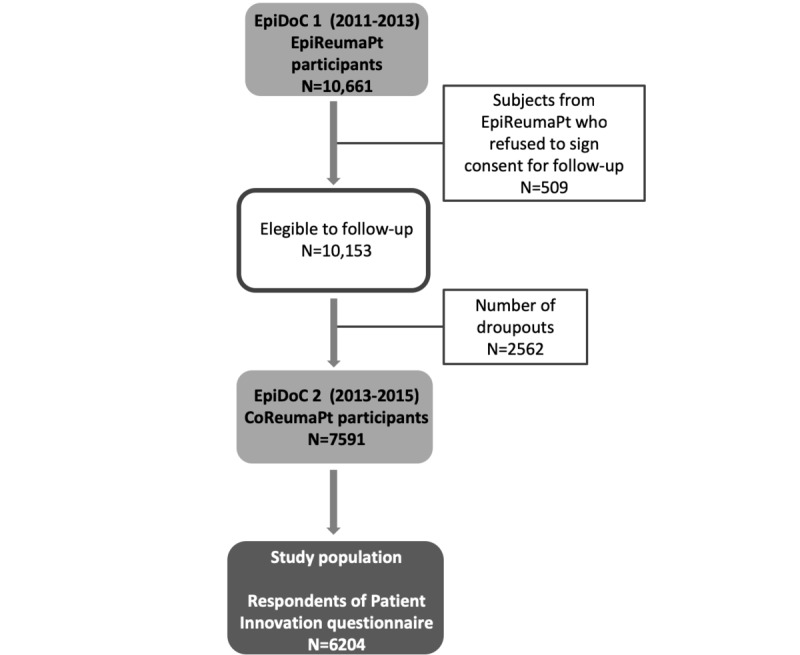
Flowchart of the population-based study named Epidemiology of Chronic Diseases (EpiDoC). The first wave (2011-2013) is entitled EpiDoC 1 - Epidemiology of rheumatic diseases study (EpiReumaPt). The second wage (2013-2015) is entitled EpiDoc 2 - Cohort of rheumatic diseases (CoReumaPt).

The innovation section of the survey, which is the focus of this paper, was integrated into a larger project, the second wave of a longitudinal, prospective, observational, population-based study named Epidemiology of Chronic Diseases (EpiDoC) (Figure 1) [18]. The first wave, entitled EpiDoC 1 Epidemiology of Rheumatic Diseases Study (EpiReumaPt), was rigorously designed to gather a representative random sample of residents in Portugal. In this phase, data were collected by face-to-face interviews of 10,661 individuals in the period from 2011 to 2013 [19]. Inclusion criteria were as follows: (1) Portuguese speaking individuals, (2) aged 18 years or older, (3) noninstitutionalized (excluding hospital or nursing homes, military barracks, and prisons), (4) for whom cognitive and physical impairments did not prevent completion of the survey, and (5) who were living in a private household in the country.

Participants were selected through a process of multistage random sampling. The sample was stratified according to the Portuguese statistic regions in the 2001 Census and the size of the population (<2000; 2000-9999; 10,000-19,999; 20,000-99,999; and ≥100,000 inhabitants). The number of participants of each stratum was proportional to the actual distribution of the population. In Madeira and the Azores, the sample size was increased (oversampling) to allow separate analyses in these regions. Candidate households were selected through a random route process; sampling points were randomly selected on the maps of each locality, where the interviewer began a systematic step count (defined for each locality according to its size), granting each household and everyone an equal probability of being chosen [19].

Most of the EpiDoc wave participants (10,153) also agreed to integrate into a prospective cohort and be contacted in the next round of surveying (EpiDoc 2)—the cohort of rheumatic diseases (CoReumaPt) wave (2013-2015). The Portuguese National Commission for Data Protection and the NOVA Medical School Ethics Committee have approved both EpiReumaPt and CoReumaPt [19]. The participants provided informed consent to participate in all phases of the study, and the study was conducted in accordance with the Declaration of Helsinki. Professional interviewers conducted all the interviews. In EpiDoC 2 (CoReumaPt), the follow-up phase, 7591 (out of 10,153) individuals completed a computer-assisted telephone survey. Of these, 6204 individuals were asked for and answered the innovation activity part of the survey. The questions about innovation activity were introduced 2 months after the launch of the follow-up study, which explains the difference in the number of respondents for this paper.

To guarantee the representativeness of the sample in relation to the Portuguese population (Mainland and *Madeira* and *Azores* islands), extrapolation weights were computed and used in statistical analysis. The weights were obtained by calibrating the extrapolation weights originally designed for the EpiDoC 1 (EpiReumaPt) sample. Participants and nonparticipants of the EpiDoC 2 (CoReumaPt) study were compared regarding their sociodemographic, socioeconomic, and health status characteristics. Weights were then adjusted based on this comparison and the stratification by statistical regions in Portugal, sex, and age groups [18].

**Table 1:** Scales, sources, and factor loadings.

Construct	Items	Factor loadings (N=6204)
Self-responsibility for health (2 items, adapted from a scale by Hibbard et al [22])	“It is me, more than any other person, who is responsible for my health and well-being.” (5-point Likert scale: 1=totally disagree, 5=totally agree)	0.9
Self-responsibility for health (2 items, adapted from a scale by Hibbard et al [22])	“The most important factor that influences my well-being and health is my active role and responsibility for my health.” (5-point Likert scale: 1=totally disagree, 5=totally agree)	0.9
Trust in medical doctor (Reduced scale proposed by Anderson et al [23])	“I trust my doctor so much that I always try to follow his/her advice.” (5-point Likert scale: 1=totally disagree, 5=totally agree)	0.9
Trust in medical doctor (Reduced scale proposed by Anderson et al [23])	“If my doctor tells me something is so, then it must be true.” (5-point Likert scale: 1=totally disagree, 5=totally agree)	0.9
Trust in medical doctor (Reduced scale proposed by Anderson et al [23])	“I feel my doctor does everything he/she should for my medical care.” (5-point Likert scale: 1=totally disagree, 5=totally agree)	0.8
Perceptions of medical science frontiers (new)	Do you believe that the medical science can treat your disease? (5-point Likert scale: 1=not at all, 5=completely trust)	0.9
Perceptions of medical science frontiers (new)	How likely is it that the medical science can successfully treat you for your disease? (5-point Likert scale: 1=not at all, 5=it certainly can)	0.9
Intention to adopt a patient-developed solution [20]	How likely is it that you would use a solution developed by another patient to help you cope with your ailment? (5-point Likert scale: 1=very unlikely, 5=I would definitely)	0.9
Intention to adopt a patient-developed solution [20]	Do you intend to use a solution developed by another patient to help you cope with your ailment? (5-point Likert scale: 1=I do not intend to use, 5=I definitely intend to use one)	0.9

### Survey Flow and 3 Groups of the Respondents

The first group of questions in the innovation section of the survey measured contextual factors, self-responsibility for health management, search for health information, the frequency of online and face-to-face interactions with peers, and trust in medical doctors and medical science. Next, respondents answered a question that split the sample into 3 groups: (1) the developers of solutions to cope with their health disorders, (2) adopters of health-related solutions developed by other patients or caregivers, and (3) the remaining population.

The question asked if an individual had developed a health-related solution or adopted a health-related solution developed by other patients. In the case of an affirmative response, the survey continued with sections that focused on the details about solution development or adoption, dividing the population into the developer or adopter groups. The third group, those who neither innovated nor adopted a solution, were asked whether they have ideas about potential solutions for health-related problems they so far encountered. Those who neither developed nor adopted a patient-developed solution were also asked about their intentions to adopt a patient-developed solution. For all the respondents, the survey started with sociodemographic questions and ended with life habits, functional, and quality of life questionnaires.

#### Creative Activity: Solution Development or Adoption of a Patient-Developed Solution

Questions regarding user innovation were built upon a questionnaire used in user innovation measurement surveys [3], adapting it to the health care context. As the conversation was phone-based, the calling party explicitly introduced the purpose of the innovation-related questions. The interviewers asked the respondents if they had, in their free time, done anything that would help them or someone close to them to cope with their health disorders. To ease the interpretation, we provided mental cues of what the potential solutions may be. The cues suggested to the respondents were medical aid instrument, medical dispositive, behavioral strategy (eg, a diet or an exercise plan), tools for everyday life at home or work, solutions related to one’s appearance, medication or a combination of drugs, and natural products.

The question about creative activity is formulated to ask about newly developed or modified solutions and about the adoption of a patient-developed solution for personal use or for someone close to the respondent. From the survey responses on this question, 2 dependent (binary) variables were created, a solution development variable (developer) and a solution adoption variable (adopter). Furthermore, the origin of the advice for the adopter was asked for, to ensure that the source of the solution is a patient/caregiver. The objective was to identify the characteristics of those who had engaged in creative activity, regardless of the artifact’s quality that is developed or adopted.

A large share of the population is likely to be neither solution developers nor adopters of peer-developed solutions, as not everyone has a need for a solution to cope with health-related issues. For this group, and to study the drivers of the attitude toward peer-developed solutions in a general population, the theory of planned behavior [20] is used. According to the theory, intentions are a relatively good proxy of future behaviors [21]. The behavioral intentions to adopt a patient-developed solution in this paper are measured using a 2-item scale (Table 1). Note that because of the survey complexity, the groups are exclusive, which means that the developers were neither asked if they had also adopted nor about their intentions to adopt a solution. Furthermore, the adopters were not asked about their intentions to adopt a solution.

#### Health Care Contextual Factors

Considering the earlier stated goal of this paper, learning about individual creative activity in the health care context, a set of questions was added to the survey. To learn about individual search efforts, the survey measured the depth of search for health information and health-related solutions as the average weekly time spent searching. As social interactions among patients may influence adoption or intentions to adopt a patient-developed solution, the interviewees were asked about the frequency of their interactions with individuals who are afflicted with the same health disorder or who share interests in the disorder; 5-point Likert scales were used to represent different levels of frequency of interactions.

To measure the perceptions of the scientific frontier, trust in medical doctors, and the attitude toward personal health management, where possible, existing scales were used (reported in Table 1).

The medical part of the survey, pertinent to the epidemiological study, included standard measurement instruments that assessed health and quality of life. In this paper, the EuroQoL-5D (EQ-5D) score [24] was used, as it is a validated, reliable, and short standard health state instrument that suits the context well. As we had 2 measures of EQ-5D score, 1 from EpiReumaPt (2011-2013) and the other from CoReumaPt (2013-2015), a variable that represents the difference between the 2 EQ-5D scores has been generated. Note that this variable is used only in the context of future activities and is included only in the model that predicts the intentions to adopt a solution. To assess who is afflicted with a health disorder, a binary variable is generated from self-reported data, indicating which individual has a clinically diagnosed chronic noncommunicable disease. The list included the following groups of diseases/health disorders: diabetes, pulmonary disease, cardiac disease, gastrointestinal disease, neurologic disease, mental disease, neoplastic disease, thyroid and parathyroid disease, and rheumatic disease. A person is considered ill if there was a report of having at least one disorder from the list (Multimedia Appendix 1).

### Statistical Analysis

Given that measurement instruments were used, the survey was pretested on 106 randomly selected interviewees. In this step, exploratory factor analysis is conducted to test whether theoretically constructed 4 factors could be identified and if there is a sufficient level of internal consistency.

Descriptive statistics are reported for the full sample (N=6204) after applying probability weights to obtain the population estimates [19,25]. Statistical software, StataCorp. 2017. Stata Statistical Software: Release 15. College Station, TX: StataCorp LLC, was used to conduct multivariate regression on survey data with the probability weights. The objective of the analysis is to explore the existence of statistically significant associations between the outcomes and independent variables. A total of 3 sets of analysis are conducted for the 3 groups. For the first 2 groups, developers and adopters, logistic regression is used to study the group differences. For the third group, the ones who neither developed nor adopted a peer-developed solution, the sample was divided in 2 subpopulations: (1) patients with at least one chronic noncommunicable disease and (2) healthy population. For both subsamples, ordinary least square models were used to explore the associations of the independent variables with the intentions to adopt. In all models, the threshold of statistical significance is set to *P*<.05.

The scales for self-responsibility for health, trust in doctors, and perceptions of medical science frontiers are included as standardized values, and the interpretation of the coefficients should be in terms of the SDs from the population mean.

## Results

### Sociodemographic Characteristics, Health Status, and Peer Interactions

The results of exploratory factor analysis on the initial sample of 106 individuals suggest that the items load well on the 4 factors, and all the factors have high internal consistency (alpha≥.7) [22]. In Table 1, factor loadings are reported for the entire sample.

Descriptive statistics are reported in Table 2 for the 3 groups (developers, adopters, and remaining population). In total, 6008 responses were included after removing responses of the individuals who could not answer the creative activity–related question. For categorical variables, the absolute count is provided, together with population estimates percentages in the brackets. For continuous variables, means and SDs are population estimates.

The results show that 1.3% of the population reported being developers and 3.3% peer-to-peer adopters. The respondents have on average 9 years of formal education (SD 4 years), and 49% reported being diagnosed with at least one noncommunicable chronic disease.

There are notable differences among the 3 groups along several dimensions. Within the developers’ group, males represent the majority (66%). Furthermore, unemployment or temporal disability/retirement among the developers (54%) is higher than that among adopters (39%) or the remaining population (35%). The developers have, on average, 1 more year of education than the adopters. For all 3 groups, interacting with peers (patients/caregivers) via the internet is rare, and the remaining population (neither developers nor adopters) are more active in that regard, with 2% more active people than that in the other 2 groups. Majority of the developers and adopters have frequent in-person interactions, 65% and 53%, respectively. The adopters have a higher number of comorbidities, 2.1 compared with 1.9 for developers and 1.5 for the rest of the population, on average. Although all 3 groups have left-skewed self-responsibility for health (4.9 out of 5), developers are more active than others, as 64% exercise regularly compared with around 40% in the other 2 groups. All 3 groups have high trust in doctors, with a marginally higher value for the remaining population, 4.5 compared with 4.3 out of 5. Perception of medical science frontier is also left-skewed, with average values of 3.5 for the adopters and 3.8 for the other 2 groups.

Absolute values of correlations between independent variables (correlation matrix available upon request) were below .35, with 4 exceptions. These exceptions were (1) age and education (*r*=−.6), (2) age and having at least one chronic disease (*r*=.41), (3) having at least one chronic disease and the quality of life score (*r*=−.38), and (4) quality of life score and education (*r*=.38). However, at these values, the correlation listed above are not considered problematic regarding multicollinearity.

**Table 2:** Descriptive statistics for the 3 groups (Innovator, Adopter, Remaining Population).

Population characteristics		Innovator (n=75)	Adopter (n=210)	Remaining population (n=5723)
Gender (female), n (%)		40 (34.0)	172 (58.4)	3146 (47.6)
Age (years), mean (SD)		44.62 (14.40)	49.53 (17.09)	46.41 (17.85)
Years of education, mean (SD)		9.36 (3.28)	8.33 (3.93)	8.92 (3.81)
**Employment status, n (%)**				
	Employed full-time, part-time, or domestic worker	38 (45.8)	116 (60.9)	3193 (65.2)
	Temporally work disabled/retired	24 (22.4)	70 (30)	1834 (24.9)
	Unemployed	8 (31.7)	23 (9.1)	479 (9.9)
**Portuguese Nomenclature of Territorial Units for Statistics, n (%)**				
	Norte	17 (33.1)	65 (32.5)	1883 (37.8)
	Centro	23 (36.4)	49 (25.4)	1210 (23.7)
	Lisboa	15 (17.2)	46 (28.2)	1135 (24.3)
	Alentejo	5 (8.2)	12 (7.4)	264 (6.3)
	Algarve	1 (2.2)	4 (3)	170 (3.6)
	Azores	7 (1.4)	17 (1.5)	521 (2.0)
	Madeira	7 (1.6)	17 (2)	540 (2.3)
Physical exercise at least once per week, n (%)		37 (63.9)	85 (40.0)	2390 (44.6)
Quality of life, EQ-5D^a^ score—CoReumaPt^b^, mean (SD)		0.71 (0.23)	0.73 (0.27)	0.80 (0.26)
EQ-5D score difference CoReumaPt^b^-EpiReumaPt^c^, mean (SD)		−0.05 (0.26)	−0.09 (0.27)	−0.05 (0.24)
Number of chronic diseases, mean (SD)		1.81 (1.64)	2.07 (2.55)	1.51 (1.71)
**Frequency of interaction with other patients or caregivers (face-to-face), n (%)**				
	Never	30 (36.2)	88 (48.8)	3181 (58.3)
	Less than once a week	12 (10.1)	61 (26.9)	1298 (22.3)
	At least once a week	33 (53.7)	60 (24.3)	1207 (19.4)
**Interaction with other patients or caregivers (internet), n (%)**				
	No	69 (92)	248 (92)	5450 (96.1)
	Yes	6 (8)	16 (8)	179 (3.9)
Depth of search: search time on health (hours per week), mean (SD)		1.97 (4.2)	1.09 (2.08)	0.47 (1.64)
Self-responsibility for health, mean (SD)		4.82 (0.28)	4.90 (0.30)	4.86 (0.41)
Trust in doctors scale, mean (SD)		4.31 (0.73)	4.35 (0.86)	4.47 (0.79)
Intentions to adopt, mean (SD)		—^d^	—	2.63 (0.3)
Perceptions of medical science frontier, mean (SD)		3.81 (0.85)	3.53 (1.12)	3.85 (0.94)

^a^EQ-5D: EuroQoL-5D.

^b^CoReumaPt: Cohort of rheumatic diseases (EpiDoC 2).

^c^EpiReumaPt: Epidemiology of rheumatic diseases study (EpiDoC 1).

^d^Not applicable.

### Results of the Multivariable Analysis

Results of the multivariable analysis are shown in Table 3. A total of 3 dependent variables (developer, adopter, and intentions to adopt) correspond to the 3 groups of interest.

#### Developer

Considering the solution development for own use (model 1), the results show that women are less likely to develop a solution for own use (OR 0.40, 95% CI 0.20-0.81; *P*<.05). Education is positively associated with the development activity (OR 1.13, 95% CI 1.03-1.24; *P*<.05), and the developers are more likely to be unemployed than employed (OR 6.45, 95% CI 2.40-17.29; *P*<.01). Considering health care contextual factors, the developers are more likely to have face-to-face interactions with other patients or caregivers (once a month or more) than no interaction (OR 4.92, 95% CI 2.20-10.99; *P*<.01). Furthermore, they are more likely to have at least one chronic, noncommunicable disease (OR 2.85, 95% CI 1.30-6.27; *P*<.01) and to search for health information more intensely than the rest of the population (OR 1.15, 95% CI 1.04-1.26).

#### Adopter

The population of adopters is significantly different from the population of developers. Adoption (model 2) is weakly positively associated with female gender (OR 1.54, 95% CI 0.94-2.52; *P*<.10) and online interactions with other patients (OR 2.12, 95% CI 0.95-4.74; *P*<.1). Adopters are, like developers, more likely to invest time to search for health information than the remaining population (OR 1.11, 95% CI 1.03-1.20; *P*<.01).

#### Remaining Population—Intentions to Adopt

In models 3 and 4, the dependent variable is the intention to adopt a solution developed by a patient or a nonprofessional caregiver. Intentions, according to the theory of planned behavior, are a proxy for actual behavior. In this paper, they are interpreted as attitudes toward peer-developed solutions.

The results for the subsample of individuals with at least one chronic noncommunicable disease (model 3) suggest a distinct combination of statistically significant associations. Intentions to adopt are negatively associated with age (beta=−.01; 95% CI −0.02 to −0.01; *P*<.01) and positively associated with education (beta=.02; 95% CI 0.00-0.03; *P*<.05). Like developers and adopters, in the remaining population, those with a chronic disease with higher intentions to adopt are more likely to invest their time to search for health-related information (beta=.07; 95% CI 0.02-0.13; *P*<.05) and more likely to have frequent in-person interactions with other patients/caregivers. Unlike the other 2 groups, developers and adopters, doctor-patient relationship plays an important role; the lower the trust in doctor, the higher is the intention to adopt a peer-developed solution (beta=−.08; 95% CI −0.14 to −0.02; *P*<.01).

Within the remaining population, the subsample of individuals without a chronic disease is very similar to the subsample of those with a chronic disease. Distinctive characteristic of the former subgroup is a negative association between retirement/temporary work disability and the intentions to adopt a peer-developed solution (beta=−.23; 95% CI −0.41 to −0.04; *P*<.05; model 4).

**Table 3:** Multivariable analysis with population estimates.

Population characteristics	Developer (versus all the others); Model 1, odds ratio (95% CI)	Adopter (versus all the others); Model 2, odds ratio (95% CI)	Intentions to adopt (remaining population with a chronic disease); Model 3, beta estimates (95% CI)	Intentions to adopt (remaining population without a chronic disease); Model 4, beta estimates (95% CI)
Gender: female versus male	*0.40^a^* (0.20 to 0.81)	1.54^b^ (0.94 to 2.52)	− *.12^a^* (−0.23 to −0.00)	0.01 (−0.10 to 0.12)
Age (years)	0.99 (0.96 to 1.02)	1.00 (0.99 to 1.02)	− *.01^c^* (−0.02 to −0.01)	− *0.01^c^* (−0.01 to −0.00)
Education (in years)	*1.13^a^* (1.03 to 1.24)	0.95 (0.87 to 1.03)	*.02^a^* (0.00 to 0.03)	*0.03^c^* (0.01 to 0.04)
Employment: temporarily work disabled/retired versus employed (full- or part-time)	1.41 (0.53 to 3.79)	0.95 (0.53 to 1.72)	−.02 (−0.16 to 0.13)	− *0.23^a^* (−0.41 to −0.04)
Employment: unemployed versus employed (full- or part-time)	*6.45^c^* (2.40 to 17.29)	1.02 (0.55 to 1.90)	.04 (−0.13 to 0.21)	−0.14 (−0.34 to 0.06)
Marital status (married or union versus single or widow or divorced)	2.46^b^ (0.98 to 6.14)	1.20 (0.66 to 2.19)	−.00 (−0.13 to 0.12)	−0.02 (−0.14 to 0.09)
Regular physical exercise	*1.87*^a^ (1.03 to 3.40)	0.85 (0.52 to 1.38)	.08 (−0.06 to 0.21)	0.03 (−0.08 to 0.14)
Health state, EQ-5D^d^ score—CoReumaPt^e^	0.63 (0.25 to 1.62)	1.09 (0.30 to 4.03)	−.02 (−0.26 to 0.23)	−0.23 (−0.61 to 0.15)
Score Difference EQ-5D score: CoReumaPt^e^-EpiReumaPt^f^	—^g^	—	−.09 (−0.31 to 0.14)	−0.03 (−0.37 to 0.32)
Face-to-face Interaction with other patients/caregivers: less than once a month versus no interactions	0.68 (0.27 to 1.71)	1.24 (0.74 to 2.08)	*.27^c^* (0.13 to 0.40)	—
F2F Interaction with other patients/caregivers: once a month or more versus no interactions	*4.92^c^* (2.20 to 10.99)	1.21 (0.72 to 2.02)	*.27^c^* (0.13 to 0.42)	—
Online interactions with other patients	0.62 (0.12 to 3.14)	2.12^b^ (0.95 to 4.74)	−.09 (−0.38 to 0.19)	—
Health information search depth (hours per week)	*1.15^c^* (1.04 to 1.26)	*1.11^c^* (1.03 to 1.20)	*.07^a^* (0.02 to 0.13)	−0.00 (−0.04 to 0.04)
Personal responsibility for health (standardized)	1.06 (0.73 to 1.56)	1.14 (0.90 to 1.44)	−.04^b^ (−0.09 to 0.00)	0.04 (−0.04 to 0.11)
Trust in physician (standardized)	0.92 (0.72 to 1.18)	0.94 (0.80 to 1.09)	− *.08^c^* (−0.14 to −0.02)	− *0.14^c^* (−0.19 to −0.09)
Perceptions of medical science frontier (standardized)	0.99 (0.71 to 1.37)	0.81 (0.63 to 1.05)	−.01 (−0.07 to 0.04)	−0.02 (−0.08 to 0.04)
With at least one disease versus no disease	*2.85^c^* (1.30 to 6.27)	1.02 (0.60 to 1.73)	—	—

^a^*P*<.05.

^b^*P*<.10.

^c^*P*<.01.

^d^EQ-5D: EuroQoL-5D.

^e^CoReumaPt - Cohort of rheumatic diseases (EpiDoC 2).

^f^EpiReumaPt – Epidemiology of rheumatic diseases study (EpiDoC 1).

^g^Not applicable.

## Discussion

### Principal Findings

The analysis suggests that solution development and adoption of peer-developed solutions are relatively infrequent but significant phenomena and that the 3 groups have distinctive characteristics. Population estimates of the share of solution developers, 1.3%, is over 2 times higher than the estimate of the share of health care–related innovation by citizens in the United Kingdom [4]. As there was no restriction on the novelty of the reported solutions, as it could not be established, the reported estimates in this paper are regarding solution developers and not user innovators. In other words, the comparison is not applicable. Regarding the population estimates of the adopters, this is the first time for such an estimate to be taken.

A series of results are aligned with the extant academic literature in user innovation. Regression results suggest that solution developers are more often men and educated individuals, confirming the findings from the study of user innovations by consumers in the United Kingdom [4]. Furthermore, our paper showed that active interactions with peers are positively associated with solution development, which corroborates the findings of Hienerth and Lettl [26] who studied the influence of peer communities on user innovation. From a public policy perspective, if the goal is to stimulate solution development by patients and caregivers in health care, a meaningful investment could be in the development of communities of peers. An example of a successful investment is the *Enabling the Future* community where patients, caregivers, and community members come together to develop open-source models of 3-dimensional-printed hands [7]. Albeit, integrating knowledge and experience of users to improve health care is complex, and the effects often fail short of the expectations [27]. Although intuition may suggest internet as a great platform for communities, the results of this paper suggest that most of the respondents, developers, adopters, and even ill people from the remaining population who have higher intentions to adopt a peer-developed solution prefer in-person interactions among peers over contacts through the internet.

Our study showed that developers are more likely to be unemployed than employed. A plausible explanation may be that they have more time to reflect upon needs and solutions or that unemployment is associated to a higher likelihood of suffering from health disorders and to having financial difficulties [23], which implies the higher need to solve problems.

Group-mean comparison suggests that the average time spent searching for health-related information by the developers (2 hours/week) is almost twice the time spent by the adopters (1.1 hours/week) and 4 times higher than that for the rest of the population (0.5 hours/week). This result emphasizes the importance of the provision of curated and accurate information, especially when, following an advice of a peer without consulting a health professional may be quite dangerous. For example, applying a plant extract without understanding side effects or permitted dosages may provoke serious health issues.

Considering adoption of solutions developed by patients or caregivers, the regression results do not suggest any stark characteristic of the group of adopters. However, the application of the intentions to adopt, a concept from the theory of planned behavior, reveals an important association. The attitude of those who did not engage in neither developing a solution nor adopting one may be influenced by the doctor-patient relationship. In light of the safety concerns regarding the diffusion of (self-made) health solutions in informal communities of patients and caregivers, doctors are a vital element of the health care system that helps patients to establish safety and efficacy of the available solutions. A negative association between the intentions to adopt and age possibly reflects the generational change in the perception of the role of the conventional health care system. In particular, older individuals may be used to the paternalistic doctor-patient relationship, and they may put a higher value on the official source of health-related solutions. Education is positively associated with the intentions to adopt a solution, which is also potentially linked to the paradigm shift in health care from paternalistic to more egalitarian relationships between patients and health professionals.

### Limitations, Strengths, and Further Research

In this paper, data have been collected from a prospective cohort; as we worked with cross-sectional data, only associations may be claimed. Recollection and interpretation bias may be present in the data. Although some people may have developed or adopted a patient-developed solution without being aware of it, the focus of this work was to explore the characteristics of those who are aware and have had chosen to develop or adopt a peer-developed solution. Hence, these biases are likely not to influence the results significantly.

A set of preemptive steps were taken before administering the survey to control for item-related (common method) bias, as suggested by Podsakoff et al [28]. These measures include ease of cognitive load on the individuals and the design of the questions and their order to avoid the item-related bias.

The advantage of the study is the size of the sample and the sampling design. As this study is conducted in 1 country, it casts doubt whether the results are generalizable to other cultural and health care policy settings.

### Conclusions

This paper is the first-of-type exploratory analysis of creative activities in the general population that focuses on health care and takes into consideration health care contextual factors. It demonstrates distinctive characteristics of: (1) the patients and caregivers who are developers of solutions, (2) the adopters of peer-developed solutions, and (3) the attitudes of the remaining population. Two actionable takeaways from the study are the importance of supplying reliable health-related information to patients who are searching and of the investment in good doctor-patient relationships.

Treating patients as equals is becoming the new mantra in organized health care systems [29], and we need to consider carefully what it means regarding their creative work, knowledge contribution, and organization and delivery of medical care. Only when we understand and support the creative contributions of the patients, we will have a system that truly integrates them.

## Appendix

Multimedia Appendix 1 Data description.

## Abbreviations

CoReumaPt: Cohort of Rheumatic Diseases
EpiDoC: Epidemiology of Chronic Diseases
EpiReumaPt: Epidemiology of Rheumatic Diseases Study
EQ-5D: EuroQoL-5D
OR: Odds ratio
